# Attention Given to Victims of Gender Violence from the Perspective of Nurses: A Qualitative Study

**DOI:** 10.3390/ijerph191912925

**Published:** 2022-10-09

**Authors:** María Dolores Ruiz-Fernández, Rocío Ortiz-Amo, Andrea Alcaraz-Córdoba, Héctor Alejandro Rodríguez-Bonilla, José Manuel Hernández-Padilla, Isabel María Fernández-Medina, María Isabel Ventura-Miranda

**Affiliations:** 1 Department of Nursing, Physiotherapy and Medicine, University of Almería, 04120 Almería, Spain; 2 Facultad de Ciencias de la Salud, Universidad Autónoma de Chile, Temuco 3580000, Chile; 3 Area of Social Work and Social Services, Department of Psychology, University of Almería, 04120 Almería, Spain; 4 Sexology and Clinical Psychology Private at Inlumine Consult, Chillán 3780000, Chile; 5 Adult, Child and Midwifery Department, School of Health and Education, Middlesex University, London NW4 4BT, UK

**Keywords:** gender violence, nurses, qualitative research, experiences

## Abstract

Women victims of gender violence consider the health system an appropriate place to seek help. Aims and Objectives: to describe and understand how nurses perceive gender-based violence in health care for women victims of gender-based violence. Qualitative descriptive study. The recommendations of the COREQ guide for qualitative research reporting were followed. Sixteen nurses who were working in different health services, both primary and specialized care, were selected to take part in the study. Three focus groups and a semi-structured interview were conducted. The ATLAS.ti 9 computer programme was used. The nurses highlighted that when caring for women who are victims of gender violence, they encounter two types of violence: invisible or latent, and visible or patent. Part of the nurses’ role is based on the skill of knowing how to act and being trained to do so. In addition, there are certain controversies around the concept and origin of violence. On the one hand, they refer to gender violence as a universal phenomenon with strong cultural and educational roots, and, on the other hand, it is difficult to characterize. The findings report how nurses play a fundamental role in caring for victims of gender-based violence, but they encounter certain difficulties regarding this concept. It is therefore essential to continuously train and educate nurses on gender-based violence. Gender violence should be a competency in nursing curricula. Health systems must offer training and awareness programmes that teach nurses to detect and make decisions regarding female victims of gender violence. It is also necessary to set up spaces in the different services so that the nurses can attend to the victims.

## 1. Introduction

Gender-based violence is defined as any act of violence based on gender, with the possible or actual result of physical, sexual, or psychological harm that includes threats, coercion, or deprivation of liberty [1]. The World Health Organization (WHO) considers it to be a serious health problem, which is related to physical and mental deterioration in women [2,3]. Likewise, it causes obstetric and gynaecological complications [4]. The most common form of gender violence is intimate partner violence. In addition, this is what has the greatest impact in the socio-healthcare field [5,6]. Gender-based violence is not caused by random and individual acts but is deeply rooted in unequal relationships between men and women [4,7], and gender inequality remains a major challenge globally [8]. Despite advances in recent years, there are still differences between men and women [9]. Violence against women is constructed as the cruellest expression of gender inequalities arising in a social system that reproduces and naturalizes the supremacy and power of men over women, presenting paths of subordination and marginalization, both in everyday life and in the public world [3,5].

In Spain, there has been an increase in the number of deaths due to gender-based violence, with a spike in 2019, reaching the highest figure since 2015, with a peak of 60 victims [10]. At the national level, there is a Government Delegation against Gender Violence. In each community there are regulations and protocols for action against gender violence. In 2015, the second edition of the Andalusian Protocol for Healthcare Action against Gender Violence was published, with the aim of joining efforts and providing health professionals with homogeneous guidelines for action in cases of violence against women [11]. The social and health sector has a primary role in the intersectoral response to violence against women [4]. Gender-based violence causes serious short-term health problems, such as injuries or anxiety. In the long term [12], this violence causes chronic pain, disability, depression, suicidal tendencies, and sexually transmitted diseases, among many other problems [2]. The health services have a duty to identify this type of violence, provide immediate care and refer to specialized services [13].

Women victims of gender violence consider the health system an appropriate place to seek help [14]. Emergency rooms and primary care centres have become the first places they resort to [15]. However, the reason why they go to the service is not always a direct injury from the aggression [13,16], but this is usually a covert manifestation, which is frequently nothing more than an instinctive way of asking for help [17].

As a number of studies show, health services are crucial to addressing this type of violence [11], and nursing professionals play a critical role in helping female victims of gender-based violence [13]. However, the literature shows that it is difficult for professionals to identify situations of abuse when there are no physical injuries [18] and that they need more training in taking a correct approach to situations of gender violence [19,20].

According to current literature, nursing as a profession dedicated to care plays a fundamental role in the commitment to equality. In addition, it is involved in the eradication of gender violence as a public health problem [7,21]. It is, therefore, necessary to carry out research from the nursing perspective on gender violence in the daily care of female victims in order to improve this basic care for victims [21]. Is for all these reasons that the objective of this study was to describe and understand how nurses perceive gender-based violence in health care for women victims of gender-based violence.

## 2. Materials and Methods

### 2.1. Design

A qualitative study with a descriptive design was carried out. This approach provides a description of the phenomenon and allows researchers to explore important questions for nursing practice, focusing on how participants see, interpret, or experience a phenomenon [22]. The recommendations of the COREQ guide for qualitative research reporting [23] were followed. “Consolidated criteria for reporting qualitative research (COREQ): a 32-item checklist for interviews and focus groups”.

### 2.2. Sample/Participants

Through convenience sampling, sixteen nurses were selected to participate in the study, with a mean age of 26.43 years (SD = 6.16). The principal investigator contacted the participants via email. The study was carried out in a province in south-eastern Spain between June and November 2020. Table 1 describes the main sociodemographic variables of the participants.

The inclusion criteria for all participants were: (1) to be a nurse and/or a care specialist nurse in either primary or specialized care; (2) to be working as a nurse at the time of the study; (3) to speak Spanish; (4) to agree to participate in the study. The following exclusion criteria were established: (1) to be carrying out teaching activities; (2) to be a nurse and be unemployed; (3) not to agree to participate in the study.

### 2.3. Data Collection

Three focus groups and a semi-structured interview were conducted by three nurses trained in gender violence with more than 5 years of care experience. They were recorded through the Blackboard Collaborate platform and then literally transcribed. Prior to conducting the focus group and the interviews, the participants were informed by the nurses of the purpose of the study, and their written consent was obtained. At the conclusion of the study, the transcripts were returned to the participants for correction or comment. Firstly, the focus groups were carried out that allowed information to be collected about topics previously incorporated in a script, and in addition, new questions that arose were added. The topics explored included the definition of the concept of gender violence, experiences, and perceptions about health intervention in violence against women, and thoughts on possible barriers to the provision of care (Table 2). The duration was about 90 min for each focus group. The semi-structured interview, which lasted 60 min, was conducted days later with open questions. The questions were the same in both the focus groups and the interview. Only one interview was conducted due to data saturation.

### 2.4. Ethical Considerations

This study has followed the 1964 Declaration of Helsinki [24] on ethical principles for biomedical research in human beings and its subsequent reforms. Informed consent was obtained from the participants, freedom of expression was guaranteed, and the participants could abandon the study at any time. They were informed of the right not to answer any question and could leave the interview at any time. All personal data obtained in this study were confidential and were treated in accordance with Organic Law 3/2018, December 5, on the Protection of Personal Data and Guarantee of digital rights [25]. The participants were informed at all times of the protection, storage, access, and subsequent destruction of the data obtained.

In the transcripts of the interviews, the confidentiality of the participants was ensured, and their names were changed to alphanumeric codes. The characteristics that might identify them and that were not relevant to the study were modified. The information obtained was used exclusively for the specific purposes of this study, in compliance with the code of good practices in research of the University of Almería (UAL, 2011). The study received the consent and approval of the Ethics Committee of the University of Almería EFM 95/2021.

### 2.5. Data Analysis

The interviews were digitally recorded and transcribed verbatim. The accuracy of the recordings was verified, and they were analysed following a thematic analysis method with the following steps [26]: (1) Familiarization with the data: by reading and rereading the participants’ transcripts; (2) Coding: the researchers selected the most significant citations and assigned them codes; (3) Creation of initial themes from the coded data: the researchers created initial themes by grouping codes that shared patterns of meaning and were related through a central idea; (4) Theme development and review: The researchers double-checked that all generated themes were consistent with the codes they included and the citations on which they were built. (5) Refine, define, and name themes: The researchers reviewed the last three themes and refined them by merging some of them. They then created the definitive names of the songs. (6) Report Writing: The researchers selected the most notable quotes and summarized the themes/sub-themes to prepare this research report. The researchers then refined the report by filtering out the essential fragments and relating them to the aims of the research and the literature review. All the information was analysed with the ATLAS.ti 9 computer programme.

### 2.6. Rigour

The quality criteria of Lincoln and Guba (1985) [27] were adopted to assess study quality. Credibility: the processes for data collection have been reproduced in detail; the interpretation was supported by the triangulation of researchers; the analysis process was reviewed by independent researchers. All the comments were accompanied by a preliminary and more detailed analysis to perfect the revision of the established codes. Transferability: a detailed description of the setting, participants, context, and method was made. Reliability: the interpretations were reviewed by a researcher from outside the study, who did not take part in the data collection. Confirmability: it was ensured that all the opinions of the participants were represented. The researchers returned the transcribed interviews and interpretations to the participants to verify their accuracy. An independent reading was carried out by the researchers to clarify and agree on the emerging themes and sub-themes.

## 3. Results

This section may be divided into subheadings. It should provide concise and precise findings from the analysis of the data. Three categories and six subcategories emerged that helped us to understand the experiences of nurses in caring for female victims of gender violence (Table 3). In addition, a selection of the most representative citations is offered in the description of the results.

### 3.1. Evidence and Indications of Gender-Based Violence in Health Care

The healthcare environment is an access route for the detection of cases of gender violence. Nurses must be alert and able to detect signs and symptoms related to gender-based violence against female victims. In addition, they should establish a relationship of professional-patient trust that provides a safe space where women can express their problems. This category reveals two subcategories where nurses report two types of violence.

#### 3.1.1. Detecting Invisible or Latent Cases of Violence

The participants said that sometimes they care for women who are victims of gender-based violence which they hide. In this type of invisible or latent violence, nurses must analyse behavioural elements of the victim, which are clues to a case of gender violence. In these cases, the physical injuries are usually superficial, and the violence is usually psychological. For this reason, the nurses argue that it is good to find separate facilities where they can explore those symptoms associated with gender violence.

*“I have been able to observe that the body language in these people is very remarkable, a sad look or eyes full of tears, participating little … These are behaviours that indicate to me as a health professional that something is not right.”*

(P1.E)

*“There are many cases in which the woman is completely annulled, I try to stay longer with the woman or have a moment of solitude or complicity to be able to talk to her.”*

(P15.GF3)

*“In some cases when I stay with them alone, I ask them if they visit their family doctor and they say no, or that they never go alone, or that they are never sick … and it is clear that these are excuses that he never leaves them alone …”*

(P2.E)

According to our participants, another element that can raise suspicions and that can be an indicator is situations in which the victim is accompanied by the aggressor, and he does not let her speak. The man’s dominance over the woman is clear. Therefore, the nurses must observe the woman’s non-verbal language, which may reveal a possible turmoil of gender violence.

*“Yes, you can notice her, especially looks, gestures, eyes looking down, half words, that they say to you when the husband is not there, and when the husband is there she does not speak to you. These kinds of things already give you a warning, as my colleague has already said, when the husband speaks over the woman and speaks in the plural, cancelling her. Just this makes you somewhat more alert.”*

(P16.GF3)

*“There are times when she has almost no access to get her purse, even the purse is under his control.”*

(P14.GF3)

#### 3.1.2. Visible or Patent Violence

According to our participants, the other type of gender violence is visible violence. Injuries and damage are seen physically, the victim recognizes it and participates in the process. In this situation of physical aggression, nurses play a fundamental role in caring for the victim. They feel essential in accompanying and supporting the battered woman, to the point that the woman is able to report the situation of abuse.

*“And, now, the other case is directly evidenced, when patients arrive who have been mistreated, with signs of violence, and they recognize it when you talk to them.”*

(P10.GF2)

*“There are also cases where the victim comes to our service looking for help and tells you what has happened… although it is very difficult for them. They need us.”*

(P1.E)

*“On some occasions women have come in beaten with something broken, which is not the first time it has happened but they have hidden it and when you get to talk to them, they admit it to you.”*

(P6.F1)

### 3.2. Role of Nurses When Confronting Gender Violence

Nurses play a fundamental role in gender-based violence as they believe that there is a high probability that female victims of gender-based violence will seek health care after suffering multiple attacks. However, the probability that they confess to being victims of gender-based violence is lower. Professionals are therefore faced with a problem that is difficult to detect. This emphasizes the importance of the correct training of healthcare personnel in medical-legal matters to correctly assist the victim. In addition, offering correct training in gender violence would influence nurses’ confidence when making a decision on witnessing signs attributable to episodes of gender violence.

#### 3.2.1. Actions of Nurses When Dealing with Gender Violence

Our participants consider that nurses must anticipate and be alert to certain behaviours or suspicious signs, which may be essential to uncover situations of gender violence. However, despite being able to detect situations of abuse, nurses do not feel prepared to act in cases of non-latent violence, such as psychological violence.

*“Nursing professionals should be aware of these types of expressions because this is what makes us aware of prior behaviour or signs of gender violence.”*

(P3.GF1)

*“We know how to act in the face of physical violence, I believe that invisible, psychological violence is worse. If what you are seeing is a bad phone call, a bad answer, authority …”*

(P14.GF3)

#### 3.2.2. Training of Nurses to Deal with Gender Violence

The participants consider that training in the detection and treatment of gender violence is a priority as facing situations of gender violence without experience or training creates doubts. The biggest problem is when dealing with cases of gender violence in which not even the victim is aware that she is a victim of gender violence.

*“Then I think that, as professionals, being trained in this is fundamental.”*

(P2.GF1)

*“On a personal level, I don’t know how to act or whether what I say to the patient is going to help her or if I’m really helping her. All this is a consequence of the lack of training, which is evidently scarce in this area, and due to inexperience”.*

(P8.GF2)

### 3.3. Controversies around the Concept and Origin of Gender Violence

When the concept of gender violence is discussed, it is sometimes complex and complicated. Nurses have different points of view on this subject, which interfere with the care they have to provide to female victims of gender violence. Our participants, when talking about the concept of gender violence, raise different aspects based on the cultural context and experiences.

#### 3.3.1. A Universal Phenomenon with Cultural and Educational Roots

The interviewees believe the concept of gender violence is linked to the characteristics of the culture to which they belong and to education. This fact further prevents the detection and care of the victims as a case that the nurses consider to be an obvious case of gender-based violence may not be so for the victim due to her culture. As a result of their life history and education, violence is normalized.

*“I think it is also a bit linked to culture because in other different cultures women might suffer violence due to the fact that men are still considered to have more rights …”*

(P15.GF3)

*“On the other hand, in this type of case that is so cultural, it is very difficult for me to get involved because I don’t know where the culture ends and violence begins, I don’t know to what extent it is normal behaviour for them, but for me it is not.”*

(P16.GF3)

#### 3.3.2. The Difficult Characterization of Gender Violence

When talking about the concept of gender violence, the participants show different nuances based on experiences and cultural context. For some nurses, the concept should be extended much further than the man/woman binomial and include homosexual couples or men as victims. For the rest, gender-based violence is associated with women because women are usually victims in heterosexual couples.

*“There is also violence against men, but there is much more against women. And other types of couples that are not heterosexual should also be looked at; in homosexual couples there is also gender violence, from man to man or woman to woman. I think we are focusing more on violence against women in a heterosexual couple because this is what is seen most and what exists most.”*

(P6.GF1)

*“I consider that gender violence is any act that can cause physical, psychological or sexual harm to women for the mere fact of belonging to the female sex.”*

(P3.GF1)

## 4. Discussion

The aim of this study was to describe and understand how nurses perceive gender-based violence in health care for women victims of gender-based violence. The analysis of the results revealed that health services can detect evidence or indications of gender-based violence in female victims [28,29] as victims resort to these services in order to seek help [2,6]. In addition, gender-based violence is considered a public health problem due to the impact it has on the health of the victim [30].

However, the participants showed difficulties in recognizing certain situations of gender violence, in addition to this being a complex concept [13,31]. They believe that gender violence can be manifested in two ways: invisible violence and visible violence. This is where the participants find themselves at an impasse when facing the concept and the evidence of whether or not there is gender-based violence, as in other studies [21,32]. As highlighted by the participants, invisible violence is very difficult to be detected, and as highlighted in other studies, invisible or latent violence is a type of violence that is covered up, with silent actions, psychologically and emotionally damaging the victim, but without apparent physical signs [33,34]. The other manifestation of violence that the participants refer to is visible violence, which is easier to detect, but, although the signs of violence can be seen as the abuser has physically harmed the victim, it is also not always easy to act. Other authors have also echoed this difficulty in acting against visible gender-based violence [33,35].

The participants perceived that they took on a fundamental role for the person being cared for, as has been observed in previous studies such as that by Loeffen (2016) [28] which highlighted the responsibility that nurses have in this area. Another aspect that the participants indicated was that they lacked training in acting against gender violence, as shown in other studies [36,37,38]. In addition, there are certain complications due to the fact that, although the nurses are sure that there is a case of gender violence, on many occasions the victim does not see it in this way. Cultural aspects of the person or feelings such as fear of the aggressor come into play [39,40].

Regarding the concept of gender violence, the nurses reflect certain controversies [41,42]. Some of the participants comment that gender-based violence should include all genders and that the concept of gender-based violence should not be understood as just violence against women [32]. However, not all of the participants agree as, for others, the concept of gender violence is the violence exerted on women through the fact of being a woman and as a result of aspects such as power inequality or gender roles [29]. As other authors point out, it is a universal fact that is rooted in culture and education, and there has always been a power relationship of subordination of men over women [10].

### Limitations

This study may have several limitations. In the first place, the variability in the professional experience of the participants can influence the perception of gender-based violence. Some of the participants had little professional experience, months, and other years. However, this variability has given us a totally different view, not only in terms of professional experience but also in terms of different healthcare settings. Secondly, the online focus groups and interviews had to be conducted online due to the health restrictions caused by COVID-19. This might have been seen as a limitation, however, it has made it possible to obtain both verbal and non-verbal information that has been used to analyse the transcripts. Lastly, the fact that the great majority of the participants were women can be considered a factor that might bias the results, but we must remember that the nursing profession consists mostly of women.

As future lines of research, it would be interesting to continue advancing and delving into the concept of gender violence from the experiences of nurses. This would help us to know what are the factors that produce this perspective and allow us to design intervention and awareness programmes adapted to nurses.

## 5. Conclusions

Because of their accessibility and frequent contact with women who are victims of gender-based violence, nurses play a fundamental role in the early detection of these cases. However, we found certain difficulties when detecting possible cases of gender violence, firstly, due to the type of violence (visible or invisible) and secondly due to the personal, educational, or cultural aspects of the victim. In addition, the concept of gender violence is complex and is understood in different ways. This all makes it difficult for the professional to care for the victim. It is essential to train nurses on issues of gender violence, starting by including the topic of gender-based violence in the nursing curriculum and providing continuous learning opportunities for nurses. In addition, health systems must give education and train nurses to make decisions on this issue and establish spaces in health centres that provide personalized and safe care to female victims of gender violence.

## Figures and Tables

**Table 1:** Sociodemographic data of the participants.

Participants	Age (years)	Gender	Profession	Work Location	Professional Experience	Training in Gender Violence	Participation
1	38	Female	Matron	Hospital Delivery Room	10 years	No	I
2	27	Female	Nurse	Primary Care	5 years	No	FG1
3	22	Female	Nurse	Internal Medicine	4 months	No	FG1
4	37	Female	Nurse	Gynaecology- obstetrics. Hospital	10 years	No	FG1
5	22	Female	Nurse	Occupational Health	9 months	Yes	FG1
6	22	Female	Nurse	Traumatology	3 months	No	FG1
7	22	Female	Nurse	Emergencies	4 months	No	FG2
8	25	Male	Nurse	Primary Care	4 months	Yes	FG2
9	22	Female	Nurse	Hospitalization Area	3 months	Basic	FG2
10	21	Female	Nurse	Hospitalization Area	4 months	Basic	FG2
11	23	Female	Nurse	Hospitalization Area	4 months	Basic	FG2
12	27	Female	Nurse	Hospital Emergencies	5 years	No	FG3
13	24	Female	Nurse	Primary Care	4 months	Yes	FG3
14	23	Female	Nurse	Hospitalization Area	5 months	No	FG3
15	29	Female	Nurse	Oncohematology	9 years	No	FG3
16	39	Female	Matron	Hospital Delivery Room	8 years	No	FG3

FG = Focus Group. I = Interview.

**Table 2:** Interview protocol.

Phase	Title	Content/Example of Questions
Introduction	Motives	Belief that your experience/opinion provides a lesson that should be known by all. I’m taking part in a study on gender violence.
	Intentions	Conduct research to publicize this experience and develop or modify protocols for prevention/care for women and girls at risk of discrimination and gender-based violence.
Beginning	Opening question	What does the term “gender violence” mean to you?
Development	Conversation guide	Tell me about any experience you have had of gender violence (in your work, environment, etc.). (How I detect it, what made you think it was that…) What does the expression “tip of the iceberg” suggest to you in terms of gender violence? What discriminatory behaviours do you believe can end in violence? How do you think that the health professional can identify cases of gender violence? What should you watch out for? What role do you think the health professional should have in the early detection of discriminatory behaviours (pre-violent)? Explain why you think that GBV (Gender-based violence) is a social problem? Why do you think it is (or not) a public health problem? Tell me about your training in gender violence (or your training needs).
Closing	Final question	Is there anything else you would like to tell me?
	Thanks. Final words	Thank you for taking part. Your answers will be of great use.

**Table 3:** Categories, subcategories, and units of meaning.

Category	Subcategory	Unit of Meaning
Evidence and indications of gender-based violence in health care	Detect cases of invisible or latent violence	Abuse of female care; anxiety; symbolic facial characteristics; control, decide for the woman; wait for the husband to leave; speak for the woman; signs of sexual abuse, fear of asking; not letting explore; not stop talking; orders
	Visible or patent violence	Blame the victim; social media; economic violence: physical violence; psychological violence; violence according to age: sexual violence
Role of the nurse when confronting gender violence	The performance of nurses when faced with gender violence	Encourage reporting; warn; coordinate with social services; leave in the hospital ward or room; refer-advise; distinguish culture-violence; social work
	Training of nurses in gender-based violence	Look for the moment; difficulty distinguishing; doubts about ability; experience; training; gain trust; want to act; rejection according to cultural identity; know how to act
Controversies around the concept and origin of gender violence	A universal phenomenon with cultural and educational roots (causes)	Social rootedness; cultural-educational; economic factors; cultural machismo; normalize; patriarchal system
	The difficult characterization of gender violence	Other violence in the family; homosexual violence; family violence: violence towards men: violence towards children; violence for being a woman

## Data Availability

Not applicable.
